# Field and Laboratory Studies for Predicting Annual Generations of *Spodoptera littoralis* (Boisd.) Using Sex Pheromone Traps and Cumulative Heat Unit Measurements in Egypt

**DOI:** 10.3390/insects17080787

**Published:** 2026-07-29

**Authors:** Ahmed M. M. Ahmed, Verónica Andrade-Yucailla, Eslam A. Y. Allam, Mohammed A. A. Saad, Samer H. Manaa, Hassan F. Dahi, M. S. Yones, Freddy Alcocer-Quishpe, Marcos Barros-Rodríguez

**Affiliations:** 1 Plant Protection Department, Faculty of Agriculture, Assiut University, Assiut 71526, Egypt; mohamed.abdelbasset@agr.aun.edu.eg (M.A.A.S.); samir.manaa@agr.aun.edu.eg (S.H.M.); 2 Centro de Investigaciones Agropecuarias, Facultad de Ciencias Agrarias, Universidad Estatal Península de Santa Elena, Santa Elena, La Libertad 204102, Ecuador; crisita_2725@hotmail.com (V.A.-Y.); falcocer1178@upse.edu.ec (F.A.-Q.); 3 Agricultural Research Center, Plant Protection Research Institute, Dokki, Giza 12611, Egypt; eessllaamm7137@gmail.com (E.A.Y.A.); hassandahi@yahoo.com (H.F.D.); 4 National Authority for Remote Sensing and Space Science, Cairo 11843, Egypt; monayones@yahoo.com; 5 Department of Animal Nutrition and Rumen Biotechnology, Ruminant Feedlot Ranch-PROCESA, Street Playita–Estero Hondo, La Maná 050202, Cotopaxi, Ecuador

**Keywords:** pheromone, *Spodoptera littoralis*, phenology, generation forecasting

## Abstract

The Egyptian cotton leafworm *Spodoptera littoralis* (Boisduval) is a major polyphagous pest in vegetable and cotton crops. Accurate forecasting of its field generations is essential for effective integrated pest management (IPM). Forecasting its generations in the field is traditionally based on the trapping of males, but the use of temperature sums (degree-days, DD) could offer a robust alternative for triggering pest control interventions. The aim of this study was to monitor the flight activity and calculate the summations of degree-days, as well as to compare expected generation peaks and the reliability of *S. littoralis* males in predicting generations over three successive growing seasons. The study was carried out over three consecutive growing seasons (2017, 2018, and 2019) at Abnob district in Assiut Governorate, Egypt, comparing twice-weekly male captures using pheromone traps and emergence dates predicted by a degree-day model established from physiological data (development threshold: 10.5 °C; thermal constant: 480.6 DD for one generation). Laboratory experiments at four constant temperatures (17, 22, 27, and 32 °C) established the lower developmental thresholds and thermal constants for each life stage: egg (t_0_ = 11.86 °C; K = 40.10 DD), larva (t_0_ = 7.56 °C; K = 283.76 DD), pupa (t_0_ = 12.27 °C; K = 149.50 DD), and pre-oviposition period (t_0_ = 12.58 °C; K = 23.96 DD). Six successive generations (observed generations) appeared after the overwintering generation each year, with the overwintering generation consistently initiating in early May each season. The capture dates from the male sex-pheromone traps coincided with the adult emergence dates calculated by the accumulated heat model; deviations between observed (the occurrence of the real peak days) and predicted generation dates ranged from −2 to +2 days in the vast majority of cases, except for in the 2nd generation of the 2019 season, when the observed generation occurred 4 days before the expected generation date. Mean accumulated thermal units per generation ranged from 479.62 to 483.78 DD across the three seasons (grand mean: 481.95 DD), with overall mean seasonal deviations of 0.00, −0.14, and −0.43 days, confirming that the model predicts generation peaks to within ±2 days in the vast majority of cases. The integration of the two methods improves the reliability of phenological forecasting for *S. littoralis*, opens new opportunities for sustainable pest suppression, and improves the timing of control actions. This combined approach is recommended as a practical decision-support tool for IPM programs targeting *S. littoralis* in Egypt and comparable Mediterranean agroecosystems.

## Simple Summary

This study evaluated the degree-day (DD) model for predicting field generations of *Spodoptera littoralis*, a destructive pest in Egypt. The objective was to monitor male flight activity and calculate thermal sums to compare expected generational peaks against those observed over three seasons (2017–2019) in Assiut. Previously established laboratory-derived thermal parameters were validated under field conditions and used for generation forecasting. The main result identified six successive annual generations following overwintering. Data from pheromone-trap captures closely matched the adult emergence predicted by the thermal model. In conclusion, the degree-day model proved to be a robust and reliable alternative for predicting generations, optimizing integrated pest management interventions.

## 1. Introduction

The Egyptian cotton leafworm *Spodoptera littoralis* (Boisduval) (Lepidoptera: Noctuidae) is a key pest in Africa [1], the Mediterranean Basin, southern Europe, Asia and tropical agroecosystems [2,3], causing severe defoliation on more than 80 plant species, including cotton, maize, soybean, tomato, and numerous vegetables, resulting in severe economic losses [3,4,5,6]. In Egypt, it is considered the main economic pest and presents one of the main obstacles threatening cotton production [7]. Because reliable methods for forecasting adult flight are limited, management of larval populations is often reactive rather than preventive.

After determining sex pheromone type, i.e., (Z,E)-9,11-tetradecadienyl acetate and (Z,E)-9,12-tetradecadienyl acetate [8], pheromone-baited traps are now commonly used to track populations of adult males [9,10]. However, pheromone effectiveness can vary depending on microclimatic factors, population density, and competition with wild females [11]. Trap-catch data can provide a direct, season-long record of male flight activity and, when properly analyzed, can reveal the commencement, timing, and relative magnitude of adult generations. However, pheromone trap catches are determined by several factors, viz., lure release rate, trap pattern, weather factors, and male longevity, which might make the direct prediction of larval infestation peaks more difficult. In parallel, phenology models based on cumulative heat units (degree-days) allow for the prediction of phenological stages based solely on the insect’s constant physiological parameters and ambient temperatures [7].

Building on this broader precedent, lepidopteran insect pest forecasting and observing population onset using pheromone traps based on cumulative heat units were reported [12,13].

Although cumulative heat-unit models and pheromone traps have been widely used in forecasting insect pest populations, limited information is available regarding the field validation of the laboratory-derived thermal requirements of *S. littoralis* under Egyptian agroclimatic conditions. Previous studies have reported developmental thresholds and thermal constants for this species; however, the practical reliability of these parameters for forecasting field generations across successive growing seasons remains insufficiently evaluated. Furthermore, to our knowledge, few studies have directly compared generation peaks predicted by degree-day accumulation models with independently observed field populations monitored using sex pheromone traps. Therefore, the present study aimed to validate previously established thermal requirements of *S. littoralis* through three consecutive growing seasons (2017–2019) and to assess the agreement between pheromone-trap monitoring and degree-day-based generation forecasting. By integrating laboratory-derived thermal parameters with field observations, this study provides a practical and reliable decision-support approach for improving phenological prediction and optimizing integrated pest management programs against this economically important pest.

## 2. Materials and Methods

### 2.1. Study Area

This study was conducted during three cotton growing seasons (2017, 2018, and 2019) in Abnob district, Assiut Governorate, Egypt (DMS coordinates: 27°16′10.56” N 31°09′3.78” E). The study area comprised about ten feddans (ca. 4.2 ha) of cotton (the local predominant and officially recommended cultivar for commercial cotton production in Assiut Governorate; *Gossypium barbadense* cv. Giza 98), managed according to conventional local agronomic procedures without insecticides application against *S. littoralis* to avoid any disturbance in natural population dynamics. No herbicides or other agrochemicals were applied within the experimental plot during the study period; weed control was carried out exclusively by manual hoeing (see Crop Management below), removing herbicide exposure as a confounding variable for moth behavior. The experimental plot was bordered by smallholder fields under mixed cultivation typical of the Abnob district, chiefly other cotton plots, together with maize and vegetable crops, representing the most likely local sources of immigrating *S. littoralis* adults.

### 2.2. Crop Management and Land Preparation

The experimental field was managed according to the standard agronomic recommendations of the Egyptian Ministry of Agriculture for cotton production: (1) The missing hills were replanted using seeds of the same cotton cultivar (Giza 98) to ensure a uniform plant stand. (2) The first irrigation (life irrigation) was applied 21 days after sowing. (3) Weed control was carried out by manual hoeing before irrigation and fertilization to remove weeds and maintain the planting rows. (4) Thinning was performed once at the second true-leaf stage, leaving the two most vigorous plants per hill. (5) Nitrogen fertilizer was applied at a rate of 60 units N per feddan in two equal splits: the first application was made after thinning with the first irrigation, and the second was applied with the subsequent irrigation. (6) Nitrogen fertilization was completed before the onset of flowering.

### 2.3. Laboratory Rearing and Experimental Design

The developmental threshold (t_0_) and thermal constant (K) values (presented in Results, Section 3.1) were originally established by Allam et al. [7]. The laboratory rearing and regression procedure summarized in Section 2.2 and Section 2.3 reproduces the method as originally used to derive these parameters and is described here for methodological completeness rather than as a newly repeated experiment for the present study.

Egg masses of *S. littoralis* were obtained from the Plant Protection Research Institute Cotton Leafworm Department, Giza, Egypt, and reared on fresh castor bean leaves (*Ricinus communis*) for at least four generations before use. Experiments were conducted under controlled conditions using four incubators maintained at constant temperatures of 17, 22, 27, and 32 °C (±1 °C) and 70 ± 5% R.H. to determine developmental rates for all life stages. The castor bean leaves were field collected from naturally growing plants near the rearing facility; no commercial leaf product was used in colony maintenance. Eggs were collected at each temperature and placed in glass jars (2.0 × 7.5 cm) in four replicates of 25 eggs per temperature and monitored daily for incubation period and embryonic development rate. Newly hatched larvae were reared individually in glass tubes (7.5 × 2.5 cm) on daily-replaced fresh castor bean leaves until pupation; larval duration and mortality were recorded. Newly formed pupae were housed individually in sealed glass tubes (2.0 × 7.5 cm) and monitored for pupal duration and weight until adult emergence. Newly emerged moths were paired (one male + one female per replicate) in glass mating cages supplied with *Nerium oleander* as an oviposition substrate and 10% sucrose solution; adult longevity, pre-oviposition period, fecundity, and fertility were recorded daily until parental death. All experiments were conducted on cotton plants at 70 ± 5% R.H. and with a photoperiod of 16:8 h L:D [7]. Within each incubator, the four replicate jars (25 eggs each) were arranged randomly on the shelf, with positions re-randomized periodically to control for any positional temperature or humidity gradient inside the incubator.

### 2.4. Calculation of Developmental Thresholds and Thermal Constants

The lower developmental threshold (t_0_) for each stage was determined by linear regression of the developmental rate (1/duration × 100) against temperature. The x-intercept of the regression line provides the theoretical temperature below which no development occurs. The thermal constant (K, degree-days) for each stage was calculated using the thermal summation equation presented in [14]. The linear regression equation applied was Y = a + bX, where Y is the developmental rate (%) and X is temperature (°C); t_0_ was accordingly calculated as the x-intercept of this regression line (t_0_ = −a/b). Developmental rate was expressed as (1/duration) × 100 to represent the percentage of development completed per day, a standard transformation that linearizes the rate–temperature relationship for regression purposes.

K = y (T − t_0_)

where K = thermal units (degree-days); y = developmental duration (days) at temperature T (°C); t_0_ = lower developmental threshold (°C). Alternatively, K was derived as K = (1/b) × 100, where b is the regression coefficient [15]. Analysis of variance was performed to test for significant differences in developmental durations among temperatures for each stage.

### 2.5. Pheromone Trapping

*S. littoralis* male moths were monitored in the field using sex pheromone traps (the synthetic sex pheromone capsules imported by the Ministry of Agriculture and Land Reclamation of Egypt for general use in all cotton cultivated areas). The main ingredient in the female *S. littoralis* sex pheromone was (Z,E)-9,11-tetradecadienyl acetate. The cap of the sex pheromone vial was hooked around 5–7 cm above the soapy water surface to work as a capturing solution. In the field, four water traps baited with the sex pheromone were dispersed randomly at the experimental area comprising ten feddans (ca. 4.2 ha). The traps were positioned in the field at a 2 m height on wooden poles that were separated into 25 cm high sections. Pheromone capsules were substituted every 4 weeks (28 days) and checked every 3–4 days, and all captured males of *S. littoralis* were counted, recorded, and removed. Trap catches were expressed as the mean number of males per trap per night. This was a locally assembled water-trap design (not a commercial funnel/delta trap). A plastic cup filled with soapy water served as the killing solution, with the pheromone lure suspended just above the water surface. Field monitoring with pheromone traps ran, in each season, from the date of the first trap check to the date of the last recorded catch: 20 April to 18 September (2017), 21 April to 27 September (2018), and 20 April to 23 September (2019), respectively. Rather than being maintained at a single fixed height, the 25 cm pole sections allowed trap height to be adjusted in steps as the cotton canopy grew taller over the season so that the trap opening remained just above canopy level throughout monitoring. The pheromone capsules were the standard synthetic lures distributed by the Ministry of Agriculture and Land Reclamation for area-wide *S. littoralis* monitoring and were not individually branded. The traps were placed well within the field, 30 m from the border of the field to minimize edge effects. The 3–4-day inspection interval and 4-week lure-replacement schedule follow common practices for lepidopteran pheromone-trap monitoring programs in the region, consistent with the interval used in Salman et al. [16] at the same study site. The mean trap catch per night was calculated as the simple arithmetic mean of the counts from the four traps on each check date; nights on which a trap could not be checked were excluded from that date’s mean rather than treated as zero catches.

### 2.6. Temperature Data

The numerical weather data (daily maximum and minimum air temperatures derived from satellite images) were obtained in cooperation with the National Authority for Remote Sensing and Space Science (NARSS) from NASA satellite images (CISL Research Data Archive (RDA)), which is responsible for a sizable and varied collection of meteorological and oceanographic observations, operational and reanalysis model outputs, and remote sensing datasets to support atmospheric and geosciences research.

The temperature data were obtained in cooperation with the National Authority for Remote Sensing and Space Science (NARSS) from NASA satellite images (CISL Research Data Archive, RDA), processed using the Advanced Research WRF (ARW) model; hourly Kelvin outputs were converted to Celsius (°C = °K − 273) before computing daily maxima and minima. No on-site weather station was available at the study area during 2017 to 2019; therefore, the satellite-derived daily maximum and minimum air temperatures used for degree-day accumulation could not be directly calibrated against ground-based measurements. All daily temperature records for the study period were complete, so no interpolation nor gap-filling was required.

The heat-unit formula used (*H* = Σ *Hj* (*Hj* = daily heat units, i.e., daily degree-days), with *Hj* defined piecewise above) is the method of Richmond et al. (1983) [17], previously cited in Section 2.7—no separate ‘single-sine’ method was used; the earlier wording was a placeholder and should be disregarded.

### 2.7. Degree-Day Model

The lower developmental threshold (t_0_ = 10.50 °C) and thermal constant (K = 480.60 DD) used in the present study were previously established under laboratory conditions by Allam et al. [7]. These parameters were subsequently applied and independently validated under field conditions during the 2017–2019 growing seasons through comparisons between predicted generation peaks and pheromone-trap observations.

The heat units (degree-days) were calculated using the daily maximum and minimum temperatures according to the methods of [17] via the following formula:

$$H=\sum H_{j}$$

where:

*H* = Number of heat units to emergence

$$H_{j}=\frac{\left(T_{max}+T_{min}\right)}{2}-C,\;if\;T_{max}>C\;and\;T_{min}>C$$

$$H_{j}=\frac{{\left(T_{max}-C\right)}^{2}}{2(T_{max}-T_{min})},\;if\;T_{max}>C\;and\;T_{min}<C$$

$$H_{j}=0,\;if\;T_{max}<C\;and\;T_{min}<C$$

*C* = Temperature threshold (t_0_).

### 2.8. Statistical Analysis

Data recorded for incubation period, larval duration, pupal duration, adult longevity, pre-oviposition period, and percentage of malformations at each constant temperature were subjected to one-way analysis of variance (ANOVA). Means were separated using the least significant difference test (LSD) at the 0.01 probability level [18]. All statistical computations were performed using the CoStat statistical software package, version 6.4 (CoHort Software, Monterey, CA, USA). CoStat was used only for the ANOVA/LSD analyses [19]. To test for inter-annual consistency, the peak-date deviation and the accumulated degree-days (DDs) of the seven generations (overwintering plus six successive generations) were compared among the three study seasons (2017–2019) using a repeated-measures ANOVA, with generation as the repeated-measures subject and season as the within-subject factor; the Friedman test was used as a non-parametric alternative when the Shapiro–Wilk test indicated departure from normality. Agreement between the field-observed accumulated DDs and the laboratory-derived thermal constant (480.60 DD) [7] was tested using a one-sample *t*-test, confirmed with the Wilcoxon signed-rank test. These analyses were performed using SciPy (v1.13) and Pingouin (v0.5) in Python (v3.12).

## 3. Results

### 3.1. Developmental Thresholds and Thermal Constants

The thermal parameters presented in Table 1 were previously established under laboratory conditions by Allam et al. [7] and are included here because they constitute the biological basis of the field-validation model used in the present study. Data presented in Table 1 summarize the lower developmental threshold (t_0_) and the thermal constant (*K*, expressed as degree-days, DDs) required to complete each developmental stage and the complete generation of *Spodoptera littoralis* (Boisd.) under four constant temperatures (17, 22, 27, and 32 °C) in Assiut Governorate, Egypt, during the 2017–2019 seasons. The egg stage had a lower developmental threshold (t_0_ = 11.86 °C) and a thermal constant of 40.10 DDs, reflecting embryonic sensitivity to low temperatures; no development occurs below this threshold. The larval stage showed the lowest developmental threshold among all stages (7.56 °C) but the highest thermal constant (283.76 DDs), accounting for approximately 59% of the total heat requirement of a complete generation.

The pupal stage occurred at *t*_0_ = 12.27 °C and required 149.50 DDs, reflecting high energy demands during metamorphosis. The pre-oviposition period had the highest threshold (12.58 °C), with only 23.96 DDs, indicating that oviposition initiation is especially temperature-sensitive, despite its brief duration. For the complete generation, *t*_0_ = 10.50 °C and = 480.60 DDs were determined; these two parameters form the cornerstone of the degree-day predictive model applied in the field component of this study. The larval stage constitutes the dominant fraction of the generation’s heat budget (~59%), while the egg and pre-oviposition stages together require only ~13%, and the pupal stage accounts for approximately 31%.

### 3.2. Flight Activity and Generation Peaks

**Figure 1:**
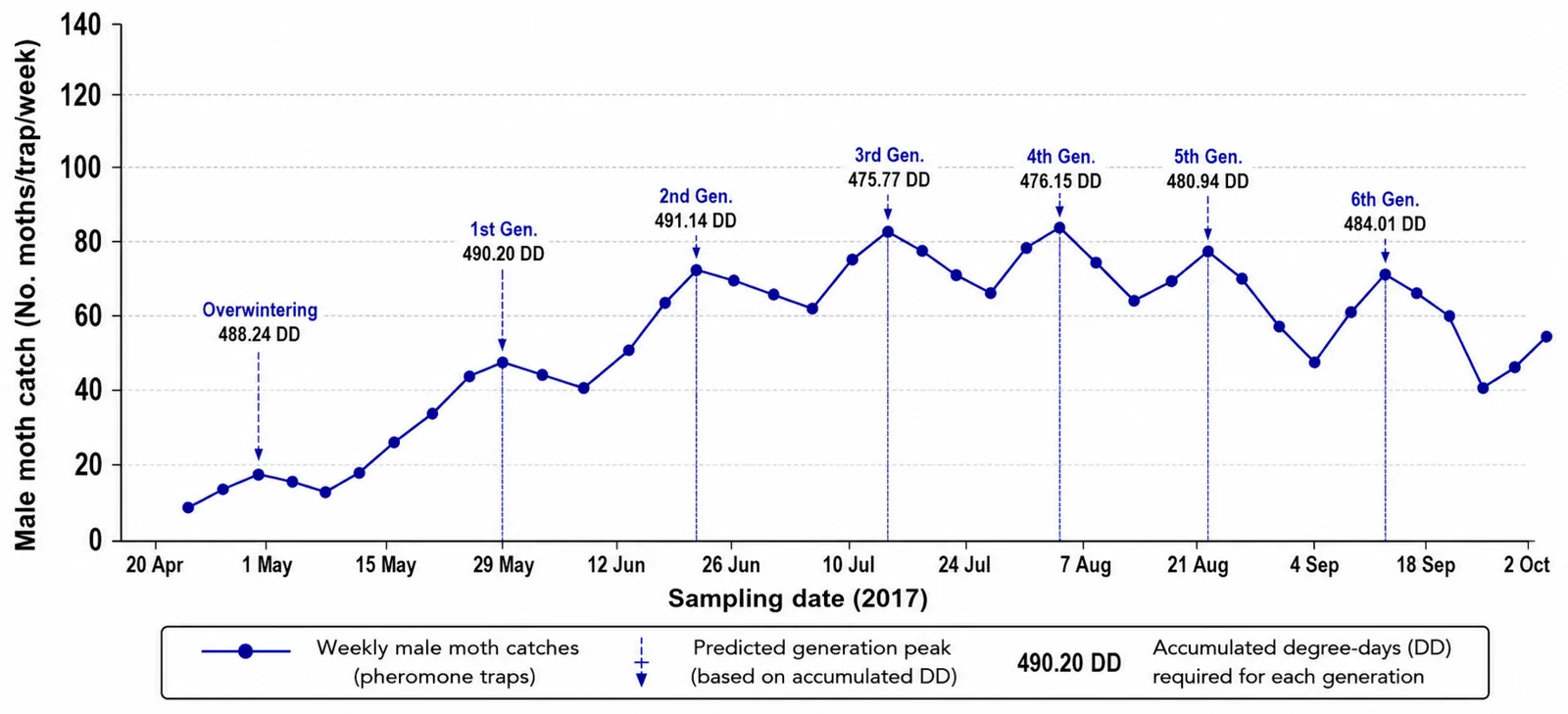
The observed and expected annual generations of S. littoralis monitored using sex pheromone traps and accumulated degree-days (DDs) on cotton plantations during 2017 at Abnob district, Assiut Governorate, Egypt. DD = degree-days accumulated above threshold temperature (t_0_ = 10.5 °C).

Pheromone trap catches revealed a consistent seasonal pattern of male flight activity across all three study seasons (2017–2019). Peak captures were recorded at discrete intervals corresponding to successive adult generations, with the overwintering generation detected in early May each year (Table 2; Figure 1). Trap-catch data were used as the biofix point for initiating degree-day accumulation, allowing direct comparison between field-observed and model-predicted generation peaks, as detailed below.

#### Expected Annual Generations of *S. littoralis* in Relation to Heat Unit Accumulations

The estimated number of thermal units needed for *S. littoralis* to complete its overwintering generation was used as a starting point to determine when the research area’s actual peak occurrence would occur at the study site in 2017, 2018, and 2019.

In the first season, 2017, six successive generations (observed generations) appeared after the overwintering generation (Table 2 and Figure 1). The 1st observed and expected generations were reported on 29 May with 490.2 DDs. The 2nd generation started on 22 June, when the expected date was 20 June, two days earlier, with 491.14 DDs. The 3rd observed generation was recorded on 13 July when the expected generation was on 12 July, one day earlier, and the thermal units were 475.77 DDs. The 4th observed generation appeared on 31 July, and the expected peak was predicted to occur on 2 August, two days later, with 476.15 DDs. The 5th observed and expected generation was reported on 24 August when thermal units were 480.94 DDs. The 6th observed generation occurred on 17 September, and the expected date was calculated as 18 September, one day later, and the thermal unit accumulation was 484.01 DDs.

**Figure 2:**
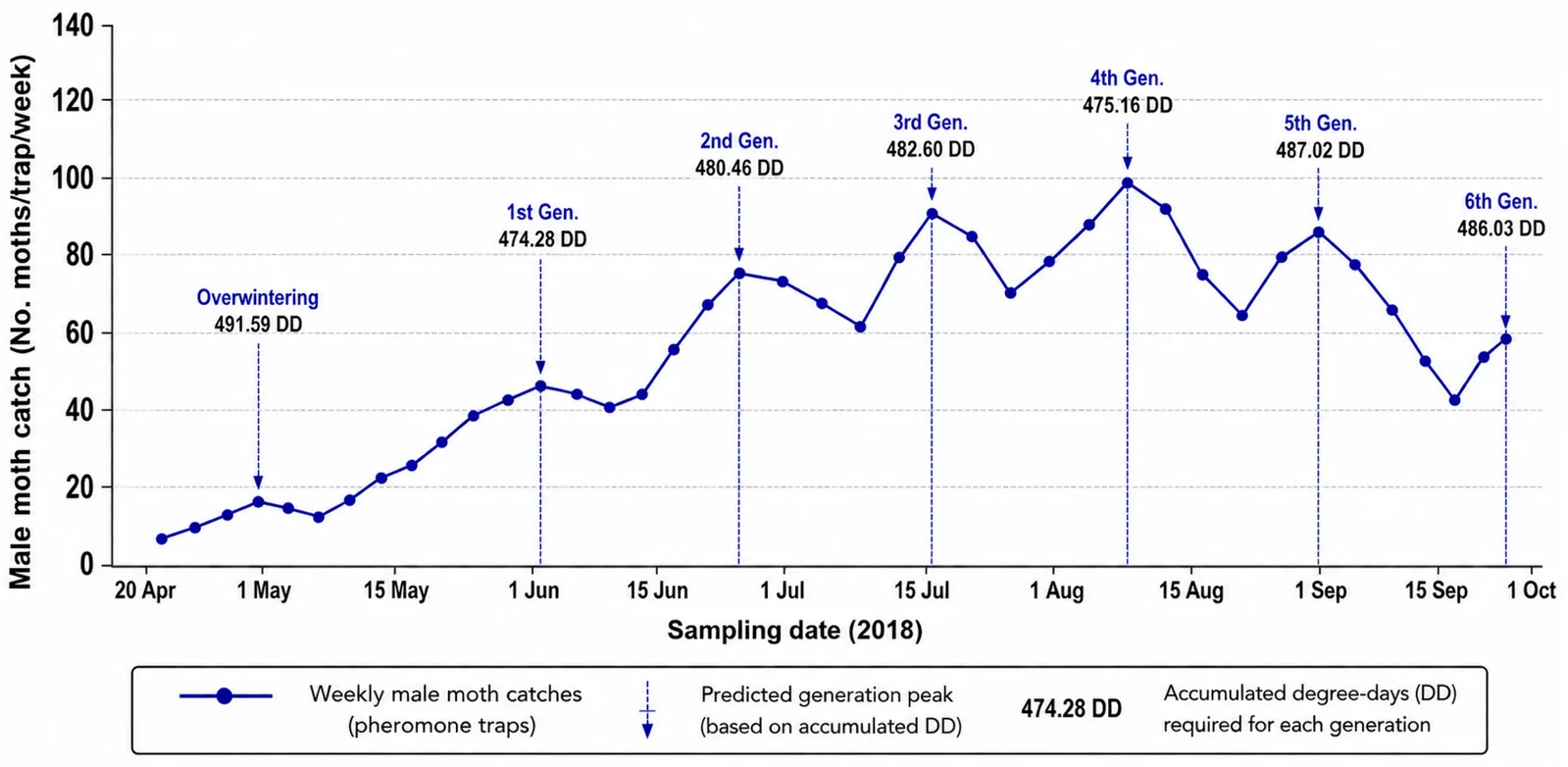
The observed and expected annual generations of S. littoralis on cotton plantations monitored using sex pheromone traps and accumulated degree-days (DDs) during 2018 at Abnob district, Assiut Governorate, Egypt. DD = degree-days accumulated above threshold temperature (t_0_ = 10.5 °C).

Data presented in Table 3 and illustrated graphically in Figure 2 for the 2018 cotton season shows the observed and expected generations. Note that the overwintering generation represented the first observed generation that occurred on 11 May, and the expected peak was set occur on the same date with 491.59 DDs.

The 1st observed generation started on 5 June, while the expected generation was 4 June, with 474.28 DDs and predicted to occur one day earlier. The 2nd observed and expected generation started on 26 June with 480.46 DDs. The 3rd observed generation started on 17 July, while the expected generation was 19 July, two days later, when the thermal units were 482.6 DDs. The 4th observed generation appeared on 10 August, and the expected generation was on 9 August, one day earlier, with 475.16 DDs. The 5th observed generation appeared on 31 August, and the expected generation was 1 September, one day later, when the thermal units were 487.02 DDs. The 6th observed and expected generations occurred on 27 September, when thermal unit accumulation was 486.03 DDs.

**Figure 3:**
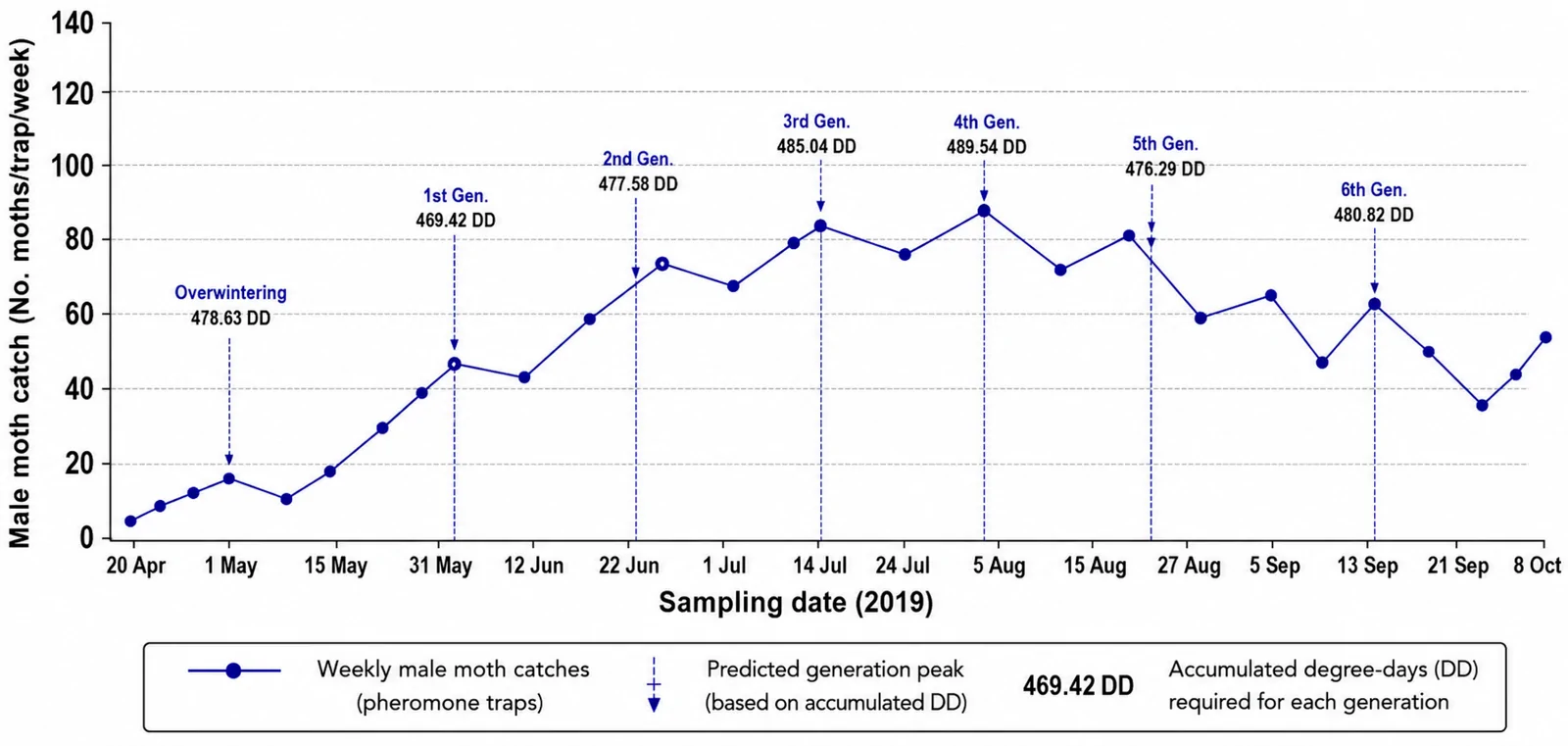
The observed and expected annual generations of S. littoralis on cotton plantations monitored using sex pheromone traps and accumulated degree-days (DDs) during 2019 at Abnob district, Assiut Governorate, Egypt. DD = degree-days accumulated above threshold temperature (t_0_ = 10.5 °C).

The data for the year 2019, listed in Table 4 and illustrated graphically in Figure 3, shows the observed and expected generations. Note that the overwintering generation represented the first observed generation that occurred on 7 May, and the expected peak was predicted to be on the same date, with 478.63 DDs.

The 1st observed and expected generation was reported on 31 May with 469.42 DDs. The 2nd observed generation started on 18 June, while the expected generation was 22 June, 4 days earlier, with 477.58 DDs. This is the largest deviation recorded across all three seasons, likely driven by an early warm episode that accelerated adult emergence ahead of the model prediction. The 3rd observed generation started on 15 July, when the expected generation was 14 July, one day earlier, when the thermal units were 485.04 DDs. The 4th observed and expected generation appeared on 5 August with 489.54 DDs. The 5th observed generation started on 26 August, and the expected generation was 27 August, one day later, when thermal units were 476.29 DDs. The 6th observed generation was recorded on 22 September, and the expected date was calculated as 21 September, one day earlier, when thermal unit accumulation was 480.82 DDs.

Data in Table 5 presents the observed and predicted field generations of *S. littoralis,* showing close agreement throughout the three study seasons. The mean accumulated thermal units required for generation completion ranged from 479.62 to 483.78 DDs. Deviations between observed and predicted generation dates were generally low and did not exceed ±2 days in most cases, except for in the 2nd generation during 2019, which showed a deviation of 4 days.

### 3.3. Statistical Comparison of Generation Timing and Degree-Day Agreement Across Seasons

Neither the peak-date deviation nor the accumulated degree-days (DDs) of the seven generations differed significantly among the three study seasons (Table 6). For deviations whose distributions departed from normality (Shapiro–Wilk W = 0.880, *p* = 0.015), the Friedman test showed no significant inter-annual difference (χ^2^ = 0.095, df = 2, *p* = 0.953); the repeated-measures ANOVA resulted in the same conclusion (F(2,12) = 0.125, *p* = 0.884). For accumulated DDs, which were normally distributed (Shapiro–Wilk W = 0.960, *p* = 0.522), the repeated-measures ANOVA likewise showed no significant seasonal difference.

(F (2,12) = 0.619, *p* = 0.555), confirmed by the Friedman test (χ^2^ = 2.00, df = 2, *p* = 0.368). The field-observed accumulated DDs (grand mean 481.95 ± 6.32 DD, n = 21 generations) did not differ significantly from the laboratory-derived thermal constant of 480.60 DD [7] (one-sample *t*-test: t(20) = 0.977, *p* = 0.340), and this agreement was confirmed at the season level (season means, n = 3: t(2) = 1.099, *p* = 0.387) and by the non-parametric Wilcoxon signed-rank test (*p* = 0.355). Together, these results provide statistical confirmation that the DD-biofix forecasting model performed consistently across the three growing seasons and that the field-validated thermal constant is statistically indistinguishable from the laboratory-derived value, supporting its direct application for field forecasting.

Regarding the 4-day advance of the 2019 s generation, comparison of the actual daily temperature record (raw meteorological data files, 2017–2019) for the pre-peak week (12–18 June) shows that 2019 was not anomalously warm relative to the same calendar window in 2017 or 2018 (mean daily maximum: 38.8 °C in 2019 vs. 42.6 °C in 2017 and 41.2 °C in 2018); therefore the previous attribution of this deviation to an unsupported “early warm episode” is not corroborated by the temperature data and thus has been removed. The 4-day advance is more plausibly explained by short-term trap-catch variability or minor asynchrony between the pheromone-trap biofix and the modeled degree-day threshold for that specific generation and should be interpreted with appropriate caution rather than as evidence of a thermal anomaly.

## 4. Discussion

The present study demonstrated that the combination of sex pheromone-trap monitoring and accumulated degree-day (DD) modeling provides a reliable and practical approach for forecasting the successive field generations of *Spodoptera littoralis* (Boisd.) under the agroclimatic conditions of Assiut Governorate, Egypt. Unlike previous studies that focused primarily on estimating thermal requirements under laboratory conditions, the present work evaluated the field performance of these parameters over three consecutive seasons and assessed their predictive accuracy using independent pheromone-trap data. These findings underscore the importance of accurately estimating the thermal units required to complete a generation of *S. littoralis* for predicting the timing and duration of each infestation, enabling timely and targeted control interventions. The narrow time intervals between expected and observed generation dates—generally ≤2 days—confirm that the combined degree-day and pheromone-trap approach provides a reliable forecast of field phenology. Because predicted peaks consistently fell within the observed window (mean seasonal deviations of 0.00, −0.14, and −0.43 days across the three seasons; Table 2, Table 3 and Table 4), growers and pest-control advisors can shorten routine monitoring periods and concentrate trap inspections around model-predicted dates. This precision reduces the cost, labor, and chemical-use risk associated with conventional calendar-based spray programs and is particularly valuable in high-infestation hotspots where early preparation of control materials is critical.

These findings concur with those determined for *S. littoralis*, *Agrotis ipsilon* by [20], *Heliothis virescens* by [12], and *Pectinophora gossypiella* by [21]. According to [22], the daily maximum and minimum temperatures account for 23% and 30%, respectively, of the population density of *S. littoralis*. Ref. [23] also claimed that *S. littoralis* had seven overlapping annual generations that began in the middle of March and continued through the beginning of November, in addition to a seventh generation that began in the second half of the following year. Several authors investigated how environmental variables and thermal unit accumulations (DDs) could be used to predict the peak moth population of *S. littoralis* [24,25,26]. In this work, the average thermal units in degree-days (DDs) needed to complete the development of *S. littoralis* generations could be expected and calculated using the daily maximum and minimum air temperatures obtained from satellite photos.

The developmental thermal threshold and thermal constant for *S. littoralis* derived in this study, i.e., 10.50 °C and 480.60 DD, respectively, were similar to those reported in the literature for *S. littoralis* development raised on an artificial diet under laboratory conditions [6], showing that the thermal requirements are broadly consistent across independent laboratory studies of this species. In addition, the good fit between observed and predicted generation peaks in this study supports the findings of [16], who calculated generation prediction for *S. littoralis* in the same study area (Abnob, Assiut Governorate), with suitable outcomes using combined heat unit accumulation and pheromone traps, where predicted peaks nearly coincided with observed captures. Cumulatively, the findings corroborate the robustness of the DD-biofix method for *S. littoralis* considering both temporal and spatial scales in Upper Egypt.

These techniques are consistent with integrated pest management (IPM) strategies recommended for other noctuids, such as *Helicoverpa armigera* and *Spodoptera exigua*, for which pheromone-based biofix and degree-day models have been successfully combined [27,28]. The simplicity of these methods, which require only a single pheromone trap and daily temperature data, makes them highly adaptable to extension programs in developing areas. This is particularly relevant in the context of projected climate change, which is expected to shift the geographic range and voltinism of *S. littoralis* across the Mediterranean Basin and beyond [1], making reliable phenological forecasting tools indispensable for future adaptive pest management strategies.

These findings indicate that the degree-day model combined with pheromone trap monitoring provides a reliable approach for forecasting field generations of *S. littoralis,* possibly improving the timing of pest management interventions. These results emphasized that calculating the thermal units needed for completing generation is very important for predicting the time of appearance and duration of generation so that the coming infestation could be controlled using various applicable methods at the right time. Generally, it is worth mentioning that the determination of the developmental threshold and accumulated thermal units (DDs) required to complete the *S. littoralis* life cycle (from egg to adult emergence) is valuable in field prediction, as determining the accumulated degree-days for the site of investigation provides a good indication of the date of oviposition (using maximum number of captured male moths that represented the actual observed peak); thus, the next generations for this pest could be expected. These results represented good prediction because the periods between the expected dates and actual observed dates were minimal, and the expected periods were before, not after, the observed dates. The accuracy of prediction according to DDs and the population patterns of *S. littoralis* in a particular area should enable growers and pest control advisors to reduce the monitoring period by allowing the checking of sex-pheromone traps during critical periods. These pest population prediction techniques are considered valuable in pest management programs. As shown in Table 2, Table 3 and Table 4, the general means of deviation in the three seasons of investigation reached 0.00, −0.14, and −0.43 before the occurrence of the real peak. This leads to perfect control and minimizes cost, time, effort, and the hazard of chemical controls usually used against this insect pest. Generally, it is beneficial for good prediction to have a positive period between the expected and actual observed dates and for this period be as short as possible to obtain good prediction accuracy according to DDs population patterns of *S. littoralis*, particularly in hot spots of infestation where early preparation of pest control materials is considered of great importance. Also, when both accumulated and calculated DDs above the threshold of development for a generation were confirmed, this technique could be considered as one of the most important factors in pest management programs.

### 4.1. Agreement Between Methods

The laboratory-derived thermal parameters presented in Table 1 provide the biological foundation for the entire degree-day modeling framework applied in this study. The lower developmental threshold for the complete generation (*t*_0_ = 10.50 °C) and the thermal constant (*K* = 480.60 DDs) established under controlled laboratory conditions (Table 1) proved robust when tested against three seasons of independent field pheromone-trap data (Table 2, Table 3 and Table 4). The fact that observed and predicted generation peaks deviated by no more than ±2 days in the vast majority of cases (Table 5) validates the use of these physiological parameters as reliable predictors of *S. littoralis* field phenology. Furthermore, the dominance of the larval stage in the heat budget (~59% of 480.60 DDs; Table 1) underscores the importance of accurate temperature-driven modeling: even small errors in threshold estimation disproportionately affect larval timing predictions, which are the most critical factors for timely pesticide interventions.

Maximum deviation did not exceed ±2 days in 2017 and 2018 and ±4 days in 2019 (2nd generation only). These results confirm that the combined pheromone-trap biofix and degree-day model reliably forecasts *S. littoralis* field generations with a ±2 days accuracy in the vast majority of cases, supporting its use as a practical IPM decision-support tool.

The fundamental core of this comparative analysis is that male lure-trap catch-peaks and degree-day generation forecasting measures are in close agreement, with deviations rarely exceeding ±2 days. This intersection demonstrates that both techniques are reasonable predictors for the dynamics of population phenology. The farmer receives an early warning of one or two days when the observed pheromone peak is ahead of the anticipated date (positive deviation). This is important, since it allows more time for control preparation. On the other hand, a negative deviation (peak earlier than expected) indicates a pesticide sprayed precisely on the expected date will encounter the pest at a slightly younger, more vulnerable stage, perhaps increasing treatment efficacy.

Compared with the work of Salman et al. [16], which reported a higher field-calibrated thermal requirement of approximately 525 DD for generation completion at the same study site (Abnob, Assiut Governorate), the present study’s mean field thermal constant (481.95 DD; Table 5) sits much closer to the laboratory-derived value of Allam et al. (480.60 DD) [7]. This closer agreement, sustained consistently across three independent seasons rather than across one or two, suggests that anchoring the degree-day model to a laboratory-validated threshold and thermal constant, combined with a fresh field biofix at each observed generation peak, yields a more precise and reproducible predictive model than does a purely field-calibrated constant estimated from a shorter monitoring period. This three-season, laboratory-anchored validation represents the main novel contribution of the present study relative to these earlier reports from the same locality.

From an IPM perspective, the integrated use of both methods addresses the inherent limitations of each technique when applied in isolation. Pheromone traps are influenced by microclimatic factors, release rate of the lure, and the longevity of males [11], whereas degree-day models depend on the precision of threshold parameters derived from laboratory experiments. The mutual cross-validation found between the two methods over three successive years (2017–2019) provides a solid basis for reliance on the prediction, especially in the critical period when decisions on pesticide treatments must be made. This integrated management strategy aligns with the recent IPM guidelines that advocate for data-driven, high-precision, low-input pest adaptability [7,16]. Furthermore, the grand mean thermal constant of 481.95 DD over the three seasons (Table 5) closely matches the laboratory-determined value of 480.60 DD [7]; thus, a strong cross-validation between controlled laboratory experiments and multi-season field observations was achieved. To attain this level of concordance between physiological time models and field phenology monitoring is an infrequent accomplishment in pest management studies and emphasizes the applicability of the present investigation as an application model for other *Spodoptera* species in the Mediterranean [1].

### 4.2. Practical Application for IPM Decision Making

In practical terms, the DD-biofix approach demonstrated here could be integrated directly into the pheromone-trap network already operated by the Egyptian Ministry of Agriculture and Land Reclamation in cotton-growing districts (Section 2.4), at negligible additional cost: field technicians who already service the traps would simply report each new peak in male catches (the biofix) to a regional extension office, which would begin accumulating degree-days from locally available temperature data. Once the running DD total approaches the thermal constant for a complete generation (480.60 DD), an advisory could be issued recommending field scouting and, if larval thresholds are confirmed, a targeted spray timed to the vulnerable early-instar larval stage before the insect begins boring into bolls or leaf whorls, rather than completed treatment on a fixed calendar schedule. Because this workflow reuses infrastructure and lure supplies that are already deployed for routine monitoring, the marginal cost to individual farmers is limited mainly to the communication channel needed to disseminate advisories (e.g., extension bulletins or SMS alerts), making this method considerably cheaper than either calendar-based spraying or maintaining on-farm weather stations.

The resilience of the method in outbreak years or under extreme/atypical weather stems from its reliance on a moving biofix rather than on a fixed calendar date: because degree-day accumulation restarts from each newly observed trap-catch peak, the model recalibrates itself each generation using real field data, which should partly buffer against the kind of short-term temperature anomalies illustrated by the 4-day deviation recorded for the second generation of the 2019 in this study. Nevertheless, this self-correction happens only after a peak has already been observed; during sudden outbreaks, or in seasons with unusually compressed or overlapping generations, the true value of the DD forecast would lie in narrowing, rather than replacing, the scouting window, and spray decisions should continue to be confirmed by field larval counts before treatment, particularly in high-risk hot spots of infestation.

## 5. Conclusions

Male sex pheromone trapping and the accumulated degree-day model are two complementary tools for forecasting generations of *Spodoptera littoralis*. While the DD model provides a more stable prediction of adult emergence, trapping offers field validation and an indispensable quantitative assessment. Their combined use significantly improves the reliability of phytosanitary decisions against this major pest.

This study is restricted to three seasons in one specific area, which is one of its limitations. It would be significant to corroborate these results using a longer study period throughout various agroecological districts, particularly in areas where movement or overlapping generations could block out the pheromone-trap signal. Future research could also immediately predict the damaging larval stages by linking the adult flight predictions to egg-laying and larval exclusion models. Concrete next steps could include multi-site trials across additional cotton-growing governorates, calibration of satellite-derived temperatures against those of ground stations, and statistical modeling of the DD–pheromone agreement across a longer time series.

## Figures and Tables

**Table 1:** Lower developmental threshold (t_0_) and degree-days (DDs) for all developmental stages and the complete generation of Spodoptera littoralis at Assiut Governorate, Egypt (2017–2019).

Developmental Stage	Lower Threshold *t*_0_ (°C)	Thermal Constant *K* (DDs)
Egg stage	11.86	40.10
Larval stage	7.56	283.76
Pupal stage	12.27	149.50
Pre-oviposition period	12.58	23.96
Complete generation	10.50	480.60

All experiments were conducted at RH 70 ± 5%, photoperiod 16:8 h L:D, on cotton plants. DDs calculated by the thermal summation equation k = y (T − t_0_).

**Table 2:** The observed and expected S. littoralis generation dates monitored using sex pheromone traps and accumulated degree-days (DDs) at Assiut Governorate during 2017.

Generations	Generation Dates		Deviation (Days)	Accumulated Degree-Days (DDs)
	Observed	Expected		
Overwintering	2/5	2/5	0.0	488.24
1st	29/5	29/5	0.0	490.2
2nd	22/6	20/6	+2	491.14
3rd	13/7	12/7	+1	475.77
4th	31/7	2/8	−2	476.15
5th	24/8	24/8	0.0	480.94
6th	17/9	18/9	−1	484.01
Average			0.0	483.78

DD = degree-days accumulated above threshold temperature (t_0_ = 10.5 °C). Overwintering generation served as biofix for initiating DD accumulation each season. Deviation = observed peak date − Expected peak date (days); positive values indicate observed peak was later than predicted; negative values indicate earlier occurrence. Mean accumulated DDs per generation (2017) = 483.78 DD; grand mean deviation = 0.00 days.

**Table 3:** The observed and expected S. littoralis generation dates monitored using sex pheromone traps and accumulated degree-days (DDs) at Assiut Governorate during 2018.

Generations	Generation Dates		Deviation (Days)	Accumulated Degree-Days (DDs)
	Observed	Expected		
Overwintering	11/5	11/5	0.0	491.59
1st	5/6	4/6	+1	474.28
2nd	26/6	26/6	0.0	480.46
3rd	17/7	19/7	−2	482.6
4th	10/8	9/8	+1	475.16
5th	31/8	1/9	−1	487.02
6th	27/9	27/9	0.0	486.03
Average			−0.14	482.45

DD = degree-days accumulated above threshold temperature (t_0_ = 10.5 °C). Overwintering generation served as biofix. Deviation = observed peak date − expected peak date (days). Mean accumulated DDs per generation (2018) = 482.45 DD; grand mean deviation = −0.14 days. The negative deviation of the 3rd generation (−2 days) indicates that the observed adult peak occurred two days earlier than predicted, likely due to above-average temperatures during that interval.

**Table 4:** The observed and expected S. littoralis generation dates monitored using sex pheromone traps and accumulated degree-days (DDs) at Assiut Governorate during 2019.

Generations	Generation Dates		Deviation (Days)	Accumulated Degree-Days (DDs)
	Observed	Expected		
Overwintering	7/5	7/5	0.0	478.63
1st	31/5	31/5	0.0	469.42
2nd	18/6	22/6	−4	477.58
3rd	15/7	14/7	+1	485.04
4th	5/8	5/8	0.0	489.54
5th	26/8	27/8	−1	476.29
6th	22/9	21/9	+1	480.82
Average			−0.43	479.62

DD = degree-days accumulated above threshold temperature (t_0_ = 10.5 °C). Overwintering generation served as biofix. Deviation = observed peak date − expected peak date (days). Mean accumulated DDs per generation (2019) = 479.62 DD; grand mean deviation = −0.43 days. The 2nd generation showed the largest deviation (−4 days), suggesting that an early warm episode accelerated adult emergence ahead of the model prediction; all other generations deviated by ≤1 day.

**Table 5:** Summary statistics of observed and predicted field generations of *Spodoptera littoralis* based on pheromone trap catches and accumulated degree-days (DDs) during the three study seasons at Assiut Governorate, Egypt.

Season	Mean DDs	Mean Deviation (Days)	Maximum Deviation	Number of Generations
2017	483.78	0.00	±2	6
2018	482.45	−0.14	±2	6
2019	479.62	−0.43	±4	6

DD = degree-days; n = 7 generations per season, including the overwintering generation. Mean deviation is the algebraic mean of all deviations per season (positive = observed later; negative = observed earlier than predicted). Three-season grand mean accumulated DDs = 481.95 DD (±2.1 SD).

**Table 6:** Statistical comparison of generation-timing deviation and accumulated degree-days (DDs) across the three study seasons (2017–2019) and agreement between field-observed DDs and the laboratory-derived thermal constant (480.60 DD).

Comparison	Test	Statistic	df/n	*p*-Value	Conclusion (α = 0.05)
Deviation (days) among seasons	Shapiro–Wilk (normality check)	W = 0.880	n = 21	0.015	Non-normal → use Friedman
Deviation (days) among seasons	Friedman test	χ^2^ = 0.095	df = 2, n = 7	0.953	No significant difference among seasons
Deviation (days) among seasons	Repeated-measures ANOVA	F = 0.125	df = 2, 12	0.884	No significant difference among seasons
Accumulated DD among seasons	Shapiro–Wilk (normality check)	W = 0.960	n = 21	0.522	Normal → RM-ANOVA valid
Accumulated DD among seasons	Repeated-measures ANOVA	F = 0.619	df = 2, 12	0.555	No significant difference among seasons
Accumulated DD among seasons	Friedman test	χ^2^ = 2.00	df = 2, n = 7	0.368	No significant difference among seasons
Field DD vs. laboratory constant (480.60 DD)	One-sample *t*-test (all 21 generations)	t = 0.977	df = 20	0.340	Field DD not significantly different from 480.60 DD
Field DD vs. laboratory constant (480.60 DD)	One-sample *t*-test (season means)	t = 1.099	df = 2	0.387	Season means not significantly different from 480.60 DD
Field DD vs. laboratory constant (480.60 DD)	Wilcoxon signed- rank test	W = 88.0	n = 21	0.355	Confirms no significant deviation from 480.60 DD

## Data Availability

The original contributions presented in this study are included in the article. Further inquiries can be directed to the corresponding authors.

## References

[1] ElShahed S.M., Mostafa Z.K., Radwan M.H., Hosni E.M. (2023). Modeling the potential global distribution of the Egyptian cotton leafworm, *Spodoptera littoralis* under climate change. Sci. Rep..

[2] EPPO *Spodoptera littoralis*; Distribution; EPPO Global Database. https://gd.eppo.int/taxon/SPODLI/distribution.

[3] Lanzoni A., Bazzocchi G.G., Reggiori F., Rama F., Sannino L., Maini S., Burgio G. (2012). *Spodoptera littoralis* male capture suppression in processing spinach using two kinds of synthetic sex-pheromone dispensers. Bull. Insectol..

[4] Campion D.G., Bettany B.W., Steedman R.A. (1974). The arrival of male moths of the cotton leaf-worm *Spodoptera littoralis* (Boisd.) (Lepidoptera, Noctuidae) at a new continuously recording pheromone trap. Bull. Entomol. Res..

[5] Hill D.S. (1983). Agricultural Insect Pests of the Tropics and Their Control.

[6] Dahi H.F. (2005). Egyptian cotton leafworm *Spodoptera littoralis* development on artificial diet in relation to heat unit requirements. Egypt. J. Agric. Res..

[7] Allam E.A.Y., Manaa S.H., Dahi H.F., Ahmed A.M.M., Yones M.S. (2021). Threshold Temperatures and Thermal Requirements of Cotton Leafworm, *Spodoptera littoralis* Hb. Egypt. Acad. J. Biol. Sci. A Entomol..

[8] Tamaki Y., Yushima T. (1974). Sex pheromone of the cotton leafworm, *Spodoptera littoralis*. J. Insect Physiol..

[9] Salama H.S. (1980). Field trapping of the cotton leafworm *Spodoptera littoralis* with its sex pheromone. Z. Angew. Entomol..

[10] El-Deeb M.A., El-Zohairy M.M., El-Sherif H. (1990). Monitoring the population fluctuations of the cotton leafworm, *Spodoptera littoralis* (Boisd.), by using sex pheromone traps. Ann. Agric. Sci. Moshtohor.

[11] El-Sayed A.M., Ganji S., Gross J., Giesen N., Rid M., Lo P.L., Kokeny A., Unelius C.R. (2021). Climate change risk to pheromone application in pest management. Sci. Nat..

[12] Potter M.F., Huber R.T., Watson T.F. (1981). Heat unit requirements for emergence of overwintering tobacco budworm, *Heliothis virescens* (F.), in Arizona. Environ. Entomol..

[13] Dahi H.F. (2007). Using heat accumulation and sex pheromone catches to predict the American bollworm, *Helicoverpa armigera* (Hub.) field generations. J. Agric. Sci. Mansoura Univ..

[14] Arnold C.Y. (1959). The determination and significance of the base temperature in a linear heat unit system. Proc. Am. Soc. Hortic. Sci..

[15] Campbell A., Mackauer M. (1975). Thermal constants for development of the pea aphid (Homoptera: Aphididae) and some of its parasites. Can. Entomol..

[16] Salman A.M.A., Dahi H.F., Fouad H.A., Elgeddawy A.M.H. (2022). Heat accumulations and sex pheromone traps as tools for predicting the cotton leaf worm *Spodoptera littoralis* (Boisd.) (Lepidoptera: Noctuidae) field generations at Assiut Governorate. J. Sohag Agrisci..

[17] Richmond J.A., Thomas H.A., Bhattacharyya H.B. (1983). Predicting Spring flight of Nantucket pine tip moth (Lepidoptera: Olethreutid) by heat unit accumulation. J. Econ. Entomol..

[18] Snedecor G.W., Cochran W.G. (1980). Statistical Methods.

[19] CoHort Software (2004). CoSta.

[20] Clement S.L., Levine E., Rings R.W. Population trends of the black cutworm correlated with thermal units accumulations. Proceedings of the IX International Congress of Plant Protection and 71st Annual Meeting of the American Phytopathological Society.

[21] Moftah E.A., Younis A.M., Girgis M.F., Khidr A.A. (1988). Thermal requirements and prediction models for pink bollworm (PBW) *Pectinophora gossypiella* (Saund.). Minia J. Agric. Res. Dev..

[22] Taman F.A. (1990). Pheromone trapping of cotton insects in relation to some climatic factors. Alex. Sci. Exch..

[23] El-Shafei S.M. (1980). Ecological Studies on the Cotton Leafworm, *Spodoptera littoralis* (Boisd.). Master’s Thesis.

[24] Hashem M.Y., Ismail I.I., Emara S.A., Dahi H.F. (1997). Seasonal fluctuations of the pink bollworm, *Pectinophora gossypiella* (Saund.) and prediction of generations in relation to heat units accumulation. Bull. Ent. Soc. Egypt.

[25] Emara S.A. Developmental biology, thermal requirements and prediction models for cotton leafworm *Spodoptera littoralis* (Boisd.) in cotton fields. Proceedings of the 2nd International Conference of Pest Control.

[26] Rashwan R.S. (2009). Predicting the Changes in the Population Density of Some Cotton Insect Pests. Ph.D. Thesis.

[27] Fye R.E., McAda W.C. (1972). Laboratory studies on the development, longevity, and fecundity of six lepidopterous pests of cotton in Arizona. USDA Tech. Bull..

[28] Shope J., Salazar-Mendoza P., Ben-Zvi Y., Rodriguez-Saona C. (2024). Refining degree-day models for *sparganothis fruitworm* in cranberry by biofix and variety. Horticulturae.

